# Preserving rural school health during the COVID-19 pandemic: Indigenous citizen scientist perspectives from a qualitative study

**DOI:** 10.3934/publichealth.2022016

**Published:** 2022-01-06

**Authors:** Prasanna Kannan, Jasmin Bhawra, Pinal Patel, Tarun Reddy Katapally

**Affiliations:** 1 Johnson Shoyama Graduate School of Public Policy, University of Regina, 2155 College Ave, Regina, SK S4P 4V5, Canada; 2 Johnson Shoyama Graduate School of Public Policy, University of Saskatchewan, 101 Diefenbaker Pl, Saskatoon, SK S7N 5B8, Canada; 3 Faculty of Health Sciences, Western University, 1151 Richmond St, London, ON N6A 5B9, UK

**Keywords:** citizen science, COVID-19, Indigenous health, mental health, qualitative, school policies

## Abstract

This qualitative study is part of Smart Indigenous Youth, a digital health community trial involving rural schools in Saskatchewan, Canada. Secondary school administrators and educators were engaged as citizen scientists in rural Indigenous communities to understand rapid decision-making processes for preserving school health during the COVID-19 pandemic, and to inform evidence-based safe school policies and practices. After COVID-19 restrictions were implemented, key informant interviews and focus groups were conducted with school administrators and educators, respectively, to understand the impact of school responses and decision-making processes. Two independent reviewers conducted thematic analyses and compared themes to reach consensus on a final shortlist. Four main themes emerged from the administrator interviews, and six main themes were identified from the educator focus group discussions which revealed a pressing need for mental health supports for students and educators. The study findings highlight the challenges faced by schools in rural and remote areas during the COVID-19 pandemic, including school closures, students' reactions to closures, measures taken by schools to preserve health during the pandemic, and different approaches to implement for future closures. Citizen scientists developed a set of recommendations, including the need for structured communication, reflection meetings, adequate funding, and external monitoring and evaluation to guide evidence-based safe school policies and practices during the pandemic.

## 1. Introduction

The coronavirus disease (COVID-19) pandemic forced global society to abruptly transform and abide by social distancing and movement restrictions. These restrictions continue to have significant impacts on the functioning of educational institutions, as well as the wellbeing of 1.6 billion students worldwide [1]–[5]. The response of educational institutions to minimize the risk of COVID-19 has ranged from remote learning and mask mandates, to social distancing and rapid testing [4],[6]. Although governments and educational institutions have considered several approaches to safely conduct in-person classes, online delivery of curricula has become commonplace for many institutions [5],[7],[8]. This haphazard combination of remote learning and incongruent safe school policies has placed an inordinate burden on administrators, educators, students, and parents/caregivers, with current evidence indicating negative consequences for student skills, learning progression, social interaction, and physical and mental health [6],[9]. Educators have also expressed concerns that school closures have had a significant detrimental impact on students' mental health and social development, with anxiety and depression rates climbing quickly [4],[7],[9]. It is clear that the COVID-19 pandemic has caused considerable disruption to education systems across the world, requiring policymakers, educators, and administrators to be responsive in rapidly adapting to changing scenarios [4],[10]. However, there are several challenges that deter safe school reopening and preservation of school health during the evolving pandemic [11]–[15].

For example, a “Back to School Study” authored by Lorenc et al.[16] highlighted that lack of trust in government guidance provided to schools was a key challenge, along with the burden of decision-making that was shifted to school administration and staff [16]. The report also identified strong school leadership and improved educator confidence and trust as important factors in safe school reopening and functioning [4],[16]. In addition, COVID-19 media coverage, particularly social media, has driven an infodemic which negatively affects mental health [17]. While there is growing evidence of the impact of COVID-19 on education systems and mental health [18], there is currently little research on how COVID-19 restrictions have impacted school health in rural areas, particularly in vulnerable Indigenous communities in settler societies such as Australia, Canada, New Zealand, and the United States [19],[20].

Indigenous Peoples' perspectives are critical to understanding how the experience and response to COVID-19 has differed across communities. Given the unprecedented nature of the COVID-19 pandemic, this study assessed the rapid adaptation of school policies and practices during the pandemic. This study, which captures Indigenous perspectives, is part of the Smart Platform, a citizen science and digital epidemiological platform for ethical population engagement, integrated knowledge translation, and policy and real-time interventions [21]. Citizen science can range from contribution (i.e., data collection) and collaboration (i.e., data interpretation) to co-creation of knowledge [22] (i.e., co-conceptualization of research and knowledge translation) by engaging citizens throughout the research process [22]–[27]. Citizen science is earning a place in national science policies [22],[28] where citizens play an active role in democratizing policy. This transformative approach [28] has particular implications for addressing societal crises such as the COVID-19 pandemic, particularly in disadvantaged rural and remote communities.

Digital citizen science can play a significant role in ethically obtaining big data to address existential crises such as the COVID-19 pandemic [29]. Digital citizen science can also support traditional scientific endeavors in rapidly responding to complex global health issues [30]. For instance, a large longitudinal cohort study (n > 50,000) which was launched in March 2020, is employing citizen science approaches to generate knowledge about participant-reported COVID-19 symptoms, behaviors, and disease occurrence [31]. Moreover, as educational systems are one of the sectors that continue to be most impacted by the COVID-19 pandemic, citizen science tools are now being developed to connect educators and students to cope with the difficulties of social isolation [32].

The purpose of this study was to engage secondary school administrators and educator citizen scientists in rural Indigenous communities in Canada to: 1) Understand rapid decision-making processes to preserve school health during the COVID-19 pandemic, including how these COVID-19 school policies influenced the health of educators and students; and 2) Inform evidence-based safe school policies and practices during this evolving pandemic. This paper describes the adaptation of a Smart Platform study—Smart Indigenous Youth—during the pandemic to collect perspectives of Indigenous school administrators and educators on school closure challenges, mental health, school leadership, and future curricula adaptations.

## 2. Materials and methods

### 2.1. Overview

This qualitative study is part of Smart Indigenous Youth [21], a 5-year mixed-methods digital community health trial that embeds culturally appropriate land-based active living programs into school curricula to promote mental health, minimize substance misuse, and prevent suicide among Indigenous youth in rural and remote First Nations Reserves in the Canadian province of Saskatchewan. A total of four schools have taken part in the Smart Indigenous Youth initiative since 2019, with all students aged 13–18 years participating in the land-based active living program. All youth and educators contributing to Smart Indigenous Youth are citizen scientists, who engage with the research team at the beginning, during, and end of each school term. This engagement is governed by the Youth Citizen Scientist Advisory Council consisting of students from the participating schools. Youth and educators provide prospective qualitative feedback via focus groups discussions, and quantitative feedback via smartphone-based surveys.

Citizen scientists informed the design, research questions and outcome measures of the Smart Indigenous Youth study. More importantly, every citizen who participated in this study had the ability to directly engage with the research team via user-triggered ecological momentary assessments on a custom-built smartphone app [33],[34]. Ecological momentary assessments provide citizen scientists' perceptions of data collection, integrated knowledge translation, and policy implications. For instance, during one of our initial data collection waves, some citizens informed us that the time-triggered surveys on smartphones expired too soon for them to respond in time. We were able to extend the expiration of time-triggered ecological momentary assessments in near-real time.

The focus on schools in First Nations Reserves for this project stems from the complex history which has put Indigenous Canadians at a higher risk of adverse physical and mental health outcomes [35]. In Canada, Indigenous Peoples consist of three groups: First Nations, Inuit, and Métis. The discriminatory categorization of Indigenous Peoples in Canada is a complicated subject, which could be perused in Smylie and Allan's report, “First Peoples, Second Class Treatment” [36]. A reserve is a piece of land allotted to First Nation bands in Canada under the Indian Act, where First Nation band members have the right to live, and band administrative and political structures are located. First Nations do not have title to reserve lands, which are held in trust for bands by the British Crown [37].

Prior to the implementation of Smart Indigenous Youth, our research team built strong partnerships with the communities which commenced by learning about their vision, priorities, and specific needs. The research team and communities identified mutual areas of interest, and built a relationship based on respect, citizen and community ownership of research data, and equity in project governance. These partnerships were established over three years during numerous in-person visits to the communities, which included meetings with leadership, Elders, youth, community members, as well as invitations to school events. The team includes researchers with a strong record of working with Indigenous communities, as well as trainees who have received extensive training in Indigenous history, cultural safety, culturally appropriate research methods, and ethics.

Since the onset of the COVID-19 pandemic, the land-based active living curricula which was administered in-person at schools participating in the Smart Indigenous Youth project had to be paused. However, the existing relationships and infrastructure of this project enabled timely modification of Smart Indigenous Youth into a qualitative study to capture the school administration's rapid response to COVID-19, as well as the subsequent decision-making processes followed to abide by public health guidelines.

### 2.2. Study design

This qualitative study used key informant interviews and focus group discussions with educator and administrator citizen scientists from two schools to capture feedback on rural schools' rapid response to COVID-19. School closures took place between March and September 2020 for the two participating schools. During this time, home learning packages were provided to students, and online engagement was conducted with some students via social media. At both schools, administration implemented safe school reopening policies and returned to in-person learning on alternate days starting October 2020. Focus group discussions took place on September 16, 2020 with one school, and October 21, 2020 for the second school.

### 2.3. Research approach

**Figure 1.**
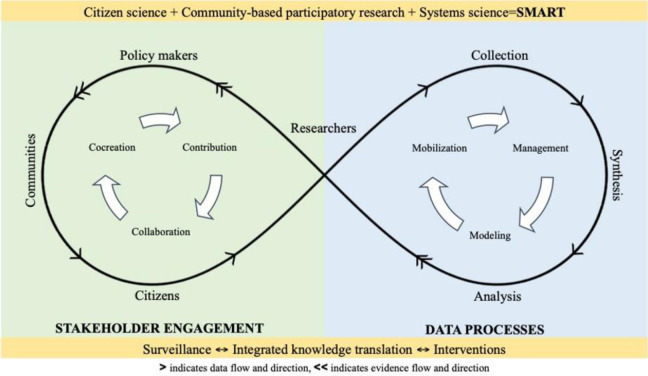
The Smart Framework: integration of citizen science, community-based participatory research, and systems science via ubiquitous tools.

Smart Indigenous Youth is part of the Smart Platform, a citizen science and digital epidemiological platform for ethical population engagement, integrated knowledge translation, and policy and real-time interventions. Although Smart Indigenous Youth is a 5-year community trial, the digital platform allowed our research team to rapidly respond to school restrictions during the COVID-19 pandemic, and meet the changing needs of our community partners and knowledge users (i.e., school administrators, educators, and youth). The initiative is informed by the Smart Framework (Figure 1) [38].

The Smart Framework integrates citizen science, community-based participatory research, and systems science to enable data collection, stakeholder engagement, and integrated knowledge translation. A cycle of contribution, collaboration, and co-creation between researchers, citizens, communities and policy makers is central to meaningful stakeholder engagement. Researchers play an important role in both citizen collaboration and knowledge translation back to all stakeholders. Together, stakeholder engagement and data processes facilitate collaborative development of research questions, data collection, and integrated knowledge translation via smartphones. Overall, the Smart Framework demonstrates how consistent engagement between researchers and citizen scientists via smartphones enables the co-creation and application of knowledge [38]. With respect to Smart Indigenous Youth, ubiquitous digital tools (e.g. smartphones) drive real-time engagement of citizen scientists in rural and remote areas, thus serving as tools of equity. Smart Framework informs the co-creation of knowledge, where youth and educator citizen scientists residing in rural and remote areas partner with researchers.

More importantly, in an effort to decolonize research methods in this study, using the Smart Framework, we have integrated citizen science with Traditional Indigenous Knowledge to ensure Two-Eyed Seeing for participatory action research [21],[39]. Decolonizing research methods requires unlearning the Western conventions of data collection, analysis, and knowledge sharing to dissociate research from its colonial European roots [25]. A Two-Eyed Seeing participatory approach allows for the thoughtful adaptation of relevant Western methodologies combined with Indigenous Knowledges and Ways of Knowing for culturally and geographically appropriate interventions.

Indigenous ceremony has been central to the design and delivery of the Smart Indigenous Youth project [40]. For instance, Elders, educators, and youth were engaged before conceptualization of the study, and played a key role in developing the engagement and data methodology [41],[42]. Elders opened Advisory Council meetings, and the team co-created knowledge in partnership with knowledge users (i.e., principals, educators, and youth), which has implications for informing and influencing culturally-relevant policies. A decolonized lens to the qualitative research method, focus groups, was taken by ensuring equitable participation of all relevant Indigenous community stakeholders, and using a virtual talking circle format to encourage open conversation and unstructured dialogue [41].

### 2.4. Ethics

Ethics approval was obtained from the Research Ethics Boards of Universities of Regina and Saskatchewan through a synchronized review protocol (REB#2017–29) [21]. To ensure respectful and culturally sensitive data collection, this research study followed relevant articles outlined in Chapter 9 of the Tri-Council Policy Statement 2 [43], the Canadian Institute of Health Research (CIHR) guidelines for working with Indigenous Peoples [44], and the principles of Ownership, Control, Access, and Possession (OCAP ®) from the First Nations Information Governance Centre [45]. The Tri-Council Policy Statement on ethical research with Indigenous communities outlines a number of important articles to conduct culturally appropriate research. The Smart Indigenous Youth project has worked closely with communities to apply these protocols, including Article 9.12: Collaborative Research, Article 9.13: Mutual Benefits in Research, Article 9.15: Recognition of the Role of Elders and Other Knowledge Holders, and Article 9.16: Privacy and Confidentiality [43]. Smart Indigenous Youth also follows CIHR guidelines for creating cultural safety, which is “a participant centered approach that encourages self-reflexivity among health researchers and practitioners... and requires building trust with Indigenous Peoples and communities in the conduct of research” [44].

Overall, the team has built strong partnerships with participating Indigenous communities, and adopted the Smart Framework [38] to co-conceptualize the project, co-create knowledge, and develop integrated knowledge translation strategies. In this study, confidentiality was ensured by de-identifying the citizen scientist personal information. All audio recordings and transcripts were coded and saved on a password-protected computer. Citizen scientists were notified that they could withdraw from the study at any time they wished. All citizen scientists provided informed consent before participation. The Standards for Reporting Qualitative Research Guidelines [46] were followed in this study.

### 2.5. Data collection

School administrators and educators were invited to participate in 60-minute semi-structured key informant interviews and focus group discussions via Zoom online meeting platform (Zoom Inc., San Jose). Interviews were digitally recorded, with the same investigator interviewing all administrators for consistency. Similarly, another investigator led all focus group discussions.

A total of 3 school administrators (2 school principals [school 1 and 2] and 1 vice-principal [school 2]) participated as administrator citizen scientists in key informant interviews. Administrator citizen scientists shared their perspectives and thought processes in developing and implementing school responses to the COVID-19 pandemic, as well as the complexities of rapid decision-making for safe school functioning. Examples of administrator questions included: “Considering the fact that the term had not ended, what steps did the school take to complete the school term?”, “How were the safe school re-opening policies and programs developed?”, “What were the barriers or challenges in policy development?”, and “Were you able to address these challenges?” (Appendix 1).

In addition, four focus groups were conducted with 19 educators from the two schools (including 4 out of 9 in school 1, and 13 out of 18 in school 2) (Table 1). Educators were split into four discussion groups with a total of 4 participants in three of the groups, and 7 in the final group. Each discussion started by asking participants to introduce themselves, their teaching grades (i.e., grade 7 educator), number of years of work experience, and the period that they worked at the school during the outbreak of COVID-19. The focus group facilitator followed a semi-structured interview guide (Appendix 2). Examples of educator questions included: “How did you cope with the lockdown?”, “What was the biggest challenge during the COVID-19 pandemic?”, and “What would you do differently if the school were to be closed again?”

### 2.6. Data analysis

A researcher transcribed all interview and focus group discussions, and created a coding manual by adopting the principles of thematic analysis. Thematic analysis is a method for systematically identifying, organizing, and offering insight into patterns of meaning across a dataset [47]. Braun & Clarke's guide [47] was followed for conducting the thematic data analysis. A researcher familiarized the data by transcribing, reading, and re-reading the transcripts. The next step included the same researcher generating a coding manual of initial themes based on a consistent pattern of key ideas. Thereafter, two researchers reviewed and fully coded the transcripts using the coding manual to verify shortlisted themes and subthemes. The same researchers reviewed themes by checking their relevance to the transcripts, and meeting online to resolve discrepancies to reach consensus on the final themes and subthemes. Qualitative research analysis software, NVIVO version 12 [48], was used for data analysis. Separate analyses were conducted for administrator interviews and educator focus group discussions for each school, and evidence across both schools was combined afterward to develop administrator and educator themes and subthemes.

**Table 1.** Sample demographics by school and gender.

Study participants	School 1	School 2
Administrators		
Principal	1	1
Vice-principal	-	1
Educators		
Group 1	4	-
Group 2	-	4
Group 3	-	7
Group 4	-	4
Total	5	17
Gender	Males: 2; Females: 3	Males: 4; Females:13

## 3. Results

Key informant interviews and focus group discussions elucidated the rapid decision-making processes that school administrators and educators had to undertake during the COVID-19 pandemic to preserve school health and ensure safe school re-openings. Key themes from administrator and educator feedback are described in separate subsections below.

### 3.1. Administrator perspectives

**Figure 2.**
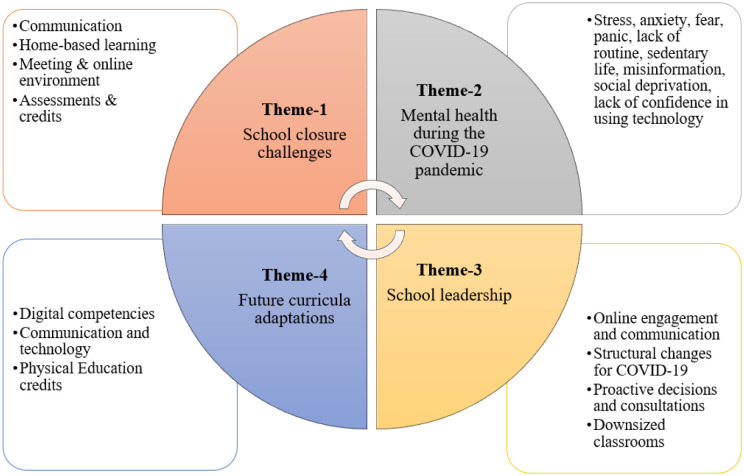
Themes and subthemes from school administrator interviews.

The results from the administrator citizen scientist engagement can be categorized into four overarching themes (school closure challenges, mental health during COVID-19, school leadership, and future curricula adaptations), with each main theme including several subthemes (Figure 2).

#### 3.1.1. School closure challenges

Administrators reported that the main challenges during school closure were related to lack of communication with students. Most students did not have consistent access to computers or internet at home, which administrators described as internet inequity. Apart from the inability to successfully facilitate online learning, internet inequity also resulted in lack of communication with students' families, which was a major concern as it limited engagement with families of students who needed mental health and social support:

*“That's why that was a little disheartening, that was the biggest complaint from teachers, is that not everybody is communicating back, not everybody is picking up their phone, like I've lost all touch. I've lost all communication with this family, and that was worrisome”* (Administrator 2).

To address the lack of online engagement with students, hardcopy learning packages were sent to students' homes in both schools. This labor-intensive approach posed additional challenges because the success rate of obtaining completed homework from students was low in both schools. The immediate school closures during the initial phase of the pandemic did not provide sufficient time for sending learning packages to students' homes, which resulted in time management issues:

*“...the biggest issue was getting things out to the students [...] we did a lot of homework packages, but then the question came upon getting them back. The teachers were more uncomfortable at the time taking the homework packages [of] the completed work, marking, and going back because of COVID threat...”* (Administrator 1).

Nevertheless, both schools continued to support students to complete assignments by using all remote communication methods possible, including phone, social networks, and emails. Frequent meetings with educators helped administrators take proactive measures for further steps; however, administrators felt that meetings during COVID-19 were onerous and increased the risk of COVID-19 outbreaks:

*“Where we met is the office and it's a small [...] table that maybe could fit 10 people around. There's, you know, 30 people standing around in this one room right by, I'd lost it. I'm like, I don't want to go to these meetings every day like their census. And so that was sort of that panic at the very beginning, not knowing what's going on, or where people have been or whatnot”* (Administrator 2).

The new online environment was considered challenging, as many of the educators reported that they were not tech-savvy and had difficulty acclimating to remote teaching methods. Similar difficulties were also faced by the students and families:

*“...there [was] some stress there, especially somebody you know some of our teachers who aren't so tech savvy, you know. All [of a] sudden being thrust to use your computer, and that might have been pretty tough.”* (Administrator 3).

School closures highlighted the difficulties that schools in rural and remote areas face due to inadequate access to digital devices and poor internet connectivity. The lack of existing infrastructure made it challenging to adapt to changing remote school protocols.

#### 3.1.2. Mental health during the COVID-19 pandemic

School administrators reported enormous mental health challenges faced by students and educators during the pandemic, which included anxiety, stress, frustration, fear of a pandemic, fear of death, and social deprivation. Misinformation about COVID-19 further exacerbated anxiety and frustration among administrators, educators, and students.

One administrator shared the fears that students had during the pandemic, which was complicated by COVID-19 misinformation:

*“...there was a little bit of that fear factor, and kids knowing – just talking – with the teachers. When we got back, the reaction with their kids or grandkids that they were looking after, the biggest thing was the miscommunication [...]the kids thought that they were going to die if they went outside. Oh yeah, I thought, wow, that's from watching the news right, and they're hearing all of this stuff on there and I'm sure parents were watching the news with them as well, but one of our high school teachers said that, yeah, her kids didn't want to go anywhere, you know, even though they were fully masked and everything. She didn't figure this out until after. Like why are you so [...] why don't you want to come with us? Why don't you want to go anywhere? Well, because I thought [it] was the fear of death”* (Administrator 2).

Overall, school administrators shared that school closures, lockdowns, and social distancing had a detrimental impact on mental health, which for many students and staff, posed a greater toll than the virus itself. While mental health was described as an existing issue in schools, the pandemic exacerbated stress and anxiety for students and educators.

#### 3.1.3. School leadership

School leadership started several initiatives to mitigate the detrimental effects of COVID-19. Online engagement was initiated with some students during the closure via Facebook and telephone contact with families. However, due to a lack of computers and/or internet access, schools struggled to consistently engage with all students:

*“So as the administrators and as the teachers, we had to make sure we were making phone calls, [...] text messages, Facebook messages, [or] emails. However, we can keep in contact with those families for the next three and a half months – we did”* (Administrator 3).

The administrators at both schools also consulted parents and educators to proactively involve them in decision-making processes, such as safe school operations. Schools made necessary infrastructure changes to facilitate social distancing, proper sanitization and other related safety measures. Schools also took the initiative to downsize classrooms by re-organizing attendance schedules:

*“...[we] alternate days on six days cycle. So half the students will come on day 1 and half will be on day 2... once you have the split yes, so here even grades 2, 4, 6, 8 will come on days 2, 4, 6 and those who have straight grades will be split and split group one and group two so group one come on odd and group two come on even days.”* (Administrator 1).

Overall, school leadership took a proactive approach to COVID-19 lockdown measures by connecting with all stakeholders (staff, educators, students, and families) in order to formulate an appropriate response. They reported working closely with all groups to make rapid changes to school infrastructure, policies, and administration of classes to ensure that students were minimally impacted.

#### 3.1.4. Future curricula adaptations

The administrator citizen scientists reported several lessons they learned from the abrupt impact of the pandemic. These lessons centered on the use of technology to bridge community gaps, and promotion of physical activity from home. School leadership teams reported working to enhance communication and technology via the provision of computers and other equipment to facilitate online learning for students:

*“I would provide a lot of opportunity to my staff in technological training. I think that is the big one, just to get familiar”* (Administrator 1).

In collaboration with the research team, one of the schools plans to award class credits for physical activity for the whole school term to address the inactivity which is perpetuated by remote work. This includes youth citizen scientists recording their daily physical activities, which will be shared with the educators on a weekly basis for awarding credits. The school leadership teams reported concern about the holistic health and wellbeing of students in an online learning environment because students' return to school full-time is uncertain:

*“So we're teaching three classes a day. But what I'd like to do as an option for those kids who want, if you want to earn an extra credit in wellness, or is that Phys Ed 20–30 getting a minimum 35 minutes of activity. A day outside for the whole school year would give us 100 credit hours. They could earn a credit and I think using the [mobile]app and some other – either video journaling or photo journaling to keep evidence of what they're doing. We might be able to earn a credit do on their own. And so with the app, like if we go to like a credit earn for outdoor Phys Ed for that stuff. The way the app is set up now, students can take a picture, or a video and do a little journal entry of some kind ....”* (Administrator 3).

School administrators recognized the need to make quick adaptations to the school curricula in an uncertain learning environment. The initial focus was on the use of technology to enable communication and promote physical activity; however, further curriculum adaptations are being discussed given the longevity of the pandemic and emerging priority areas.

### 3.2. Educator perceptions

**Figure 3.**
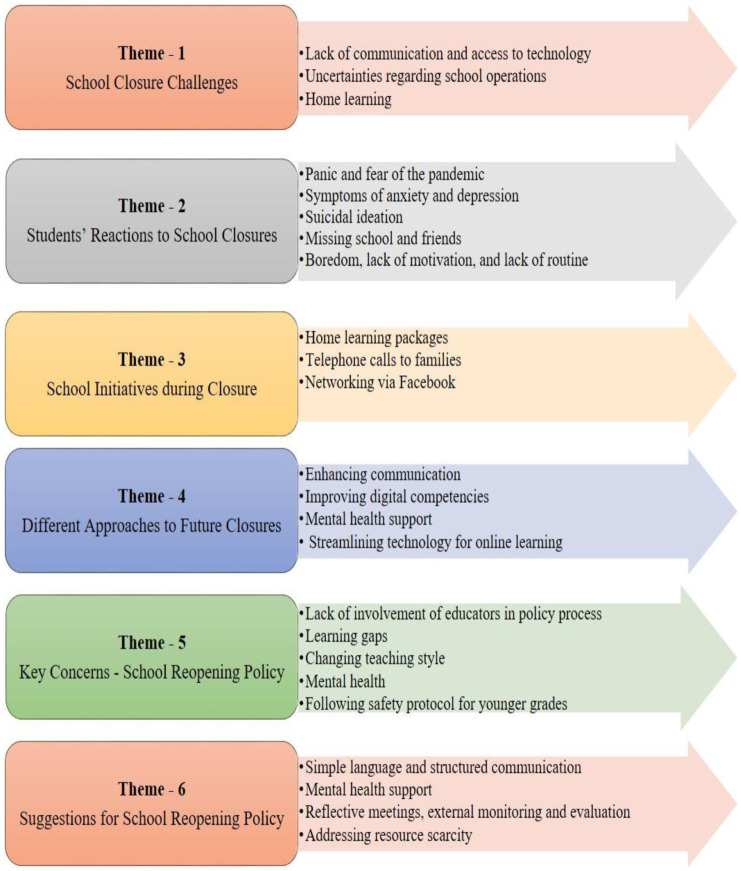
Themes and sub-themes from educator focus groups.

Discussions with educators about COVID-19 school closure challenges focused on five main themes (Figure 3) including school closure challenges, student reaction to the school closures, school initiatives during closure, different approaches to future closures, and school reopening policy.

#### 3.2.1. School closure challenges

School closures posed a range of challenges for educators and students. The key challenges educators described centered on discussions around internet access and use of technology. Educators expressed their concerns regarding the inability to engage with students due to the lack of computers and internet access for students. Moreover, they expressed challenges specific to remote learning, such as not getting back the schoolwork sent to students, which corroborated the administrators' input:

*“I guess the biggest challenge was just to maintain contact with the kids, the younger ones, to keep them motivated to continue learning at home.”* (Educator 5)

Educators also described challenges with adapting content for online classes because of lack of previous experience and training, uncertainties, and student mental health issues:

*“I had to kind of keep up with the technology and learn some new ways of communicating with other staff members. So, it was challenge, for sure.”* (Educator 6)

*“And the biggest challenge was [...] that it's tough to teach virtually, it's tough to teach language class. The kids are great. They know it's difficult, and especially if kids didn't have access to technology.”* (Educator 16)

Educators were motivated to overcome numerous challenges with online learning to ensure the success of their students; however, they felt little control over the response to mental health due to challenges with remote communication.

#### 3.2.2. Student reaction to school closures

Educators noticed a range of reactions to school closures from students, with the majority experiencing stress around the uncertainty that the pandemic created. Educators reported that many students and educators experienced a wide range of mental health issues during the pandemic, including symptoms of anxiety and depression, lack of motivation, and suicidal ideation during the lockdown which required urgent mental health interventions:

*“My biggest challenge again goes back to [...], I guess for me personally, I struggled with my teenagers. [I] just didn't really cope during that time at all because everything was closed, I couldn't get the help that I needed at that time. And my 14-year-old um, during the middle of this, ended up trying to take his own life. So, it was very, very tough because I couldn't take him to a counsellor, like I couldn't do a lot of like things that he needed. And so, being at home and not being a professional in that area was a big struggle for me because I didn't know how to. I didn't know if like, I always second guess like am I doing the right thing? Maybe this isn't what he needs.”* (Educator 6)

Mental health issues resulted from the general stress of living under pandemic restrictions, and were exacerbated by removing routine activities including attending school and having the opportunity to interact with friends and teachers.

#### 3.2.3. School initiatives during closure

Several initiatives were started during the school closure to help students and families cope with remote learning. Educators reported sending learning materials home along with hot lunches to help students cope with the pandemic (Educator 5, 6). The educators also called families, networked with some students via Facebook, and reached out to them through peer networks to ensure that students felt supported during this difficult time:

*“...so basically what we ended up doing was like we said before we had the home packages, online learning, teachers created Facebook pages uh, they created videos, and posted them on those pages. As much communication as we could have with the parents and the community, that was really encouraged, we wanted to make that we knew our kids were okay”* (Educator 8).

Despite various initiatives taken by the school to engage students during the school closures, educators reported that students continued to experience significant mental health challenges.

#### 3.2.4. Different approaches to future closures

Given their experiences with shifting to remote learning and engagement, educator citizens scientists reported several areas where they planned to respond differently for future closures. Educators felt the need to enhance communication with staff, parents, and students as policies changed. Educators stated that increasing communication could also decrease the stress and anxiety that resulted from uncertainty:

*“I think the biggest challenge is just a lot of uncertainty.”* (Educator 18)

In addition, educators were keen to improve digital competencies and streamline technology for online learning. Many educators and students were not well-versed with online teaching and learning, respectively, which affected curricula delivery. Educators shared that planning ahead and investing in online learning could potentially minimize the challenges educators and students faced.

Educators also felt the need for mental health support to cope with pandemic restrictions. In addition to measures previously described, dedicated support in coping with stress, anxiety, and depression caused by the uncertainty and isolation could help educators better perform in their jobs and aid students dealing with similar challenges.

Overall, educators were open to different approaches moving forward, particularly with regards to adopting online curricula and building mental health supports, which aligned with the administrators' perspectives on adapting future school curricula.

#### 3.2.5. Safe school reopening policy

Safe school reopening was a primary topic of concern for educators as they planned for return to in-person classes. Specific concerns that educators raised included lack of involvement in the planning process for school re-opening. They felt that their role was minimal, such as editing the information handouts for the families, rather than participating in the policy-making process. Furthermore, implementing safety protocols for younger children was identified as one of the key problems along with difficulties adapting to online curricula and remote teaching methods. While educators reported being open to curricula adaptations, they lacked necessary training:

*“I'm stressing over, how do I be creative in trying to teach or change my teaching style uh, um, to meet the COVID standards um, for the school so it's a little frustrating um, trying to be creative.”* (Educator 12)

Educators also expressed significant mental health challenges with safe school reopening, including symptoms of anxiety and depression, depression, and fear of the virus. Nevertheless, educators also came up with suggestions for safe school reopening such as usage of simple instructions for students' families and providing mental health supports for students, families, and teachers:

*“I would like somebody that works in mental health, to come online and put all the students online for my classroom and kind of talk with them about lockdown and some other things that you're going through. Like I said, some of my students have anxiety, now I guess, it wasn't as common before like and now they are getting very anxious*. (Educator 1)

Finally, educators also suggested incorporating reflective meetings, external monitoring and evaluation, and addressing resource scarcity to ensure safe school operations:

*“I think it's important to address those issues as they come up. Um, therefore I do believe in those reflective meetings because the whole thing is about the team and coming together and playing your part within that team um and so that we can all keep safe.”* (Educator 5)

Educator perceptions consistently aligned with administrator perceptions, while providing more nuance to school closure challenges, student and educator mental health crises, and curricula adaptations. However, there seemed to be disagreement in terms of participation of educators in policy making, largely due to the circumstance of rapid decision-making process which was necessary to not only implement safe school policies, but also adapt to the changing nature of public health guidelines. Nevertheless, educator suggestions could enhance the administrator decision-making processes in the future, a key knowledge translation factor of this study.

## 4. Discussion

The Provincial Government of Saskatchewan, Canada, announced school closures in mid-March, 2020 due to the emergence of the COVID-19 pandemic [49]. Rates of COVID-19 among some Indigenous communities continues to be high [50],[51], and is of concern given the numerous challenges faced by Indigenous communities (i.e., geographically rural, remote or northern locations; lack of access to clean drinking water, overcrowding, food insecurity) which exacerbates both COVID-19 risk and mental distress [52].

All schools in the province were closed indefinitely until permission was given to resume learning in-person. Schools across the nation worked to develop safe school re-opening policies [53]. Eventually, school divisions in First Nations Reserves were given the independence to develop their own safe school operation policies as long as they met the provincial public health guidelines [54],[55]. While schools globally have experienced similar challenges with remote learning and student wellness [39],[43], these issues are of special concern in rural Indigenous communities due to the lack of adequate infrastructure, digital connectivity, and access to healthcare.

Discussions with school administrators and educators in rural Indigenous communities suggest several key implications for research, policy and practice, with a particular focus on rapid responses to societal crises, school mental health supports beyond the pandemic, and re-imagining school curricula in this digital age.

### 4.1. School mental health policy

School closures due to the COVID-19 pandemic have adversely affected children and families, and placed an excessive burden on educational institutions around the world [6],[39],[43]. Our study results revealed how prolonged school closures and lack of access to technology and connectivity affected the mental health and well-being of students and educators, which corroborates existing evidence [4],[56]. As schools are also venues for non-academic supports to students by providing physical activity access [57],[58] mental health services [59], and food supplementation sources [60], it is essential to invest in school policies that preserve holistic health [61]. It is evident that substantial mental health supports are required for both educators and students to not only cope with the current pandemic, but also for future health crises [62].

Our findings suggest that the lack of mental health supports to cope with COVID-19 challenges had negative impacts on the mental health of both students and educators. The reporting of COVID-19-related symptoms of depression and anxiety, uncertainties and misconceptions, and particularly suicidal ideation require real-time interventions [62],[63]. It is important to note that while mental health was a concern in schools prior to the pandemic, the stress associated with COVID-19 exacerbated mental health issues in rural Indigenous schools. Schools reported a keen interest in developing a robust response to mental wellness. Embedding digital health solutions into school policies, especially in rural and remote areas, provides an opportunity to engage with youth and educators in real-time to minimize risk and provide appropriate supports [21],[64]. However, it is critical to address internet inequity [38],[65], which was highly prevalent among students in this study, particularly due to lack of internet access at home. As part of the Smart Indigenous Youth initiative, we were able to provide WiFi access to student citizen scientists within school premises, before, during, and after school hours. We are in the process of budgeting for data plans to provide continuous internet access to students, but it is imperative that equitable internet access becomes part of governmental policy to address this human right in the 21st century [66].

### 4.2. Curricula development and implementation

School curricula can play a critical role in mitigating significant challenges highlighted by the COVID-19 pandemic, particularly in addressing mental health issues. Evidence indicates that access to nature and time spent on the land are sources for improving health and resilience among Indigenous youth [66]. As this study is part of the Smart Indigenous Youth initiative that embeds a culturally-appropriate land-based active living program into school curricula to improve mental health outcomes [21], our findings also revealed that due to the pandemic, lack of access to land-based activities for active living in schools contributed to a decline in the mental health among Indigenous youth.

Evidence also indicates that the COVID-19 pandemic has had a particular impact on Indigenous Peoples' mental health [35],[67] and it is important to re-invest in bolstering school curricula with culturally-appropriate land-based activities which can mitigate the impact of COVID-19. COVID-19 school restrictions have also increased behavioral risk factors for non-communicable diseases, such as physical inactivity, changes in sleeping patterns, and unhealthy diet and substance misuse issues [68], reiterating the importance of adapting curricula to promote holistic health when students return to school so that the negative effects of this pandemic can be reversed. However, in the interim, while schools are functioning remotely, it is imperative to seriously consider digital technologies to enhance curricula that integrate mental health and suicide prevention [69]. This study showed that in developing sustainable curricula beyond COVID-19 [70], administrator and educator citizen identified three key methods: 1) building digital competencies, 2) increasing availability of computers and the internet at student residences to promote online learning, and 3) motivating students through physical education credits.

### 4.3. Strengths and limitations

The primary strength of this study is its ability to leverage an ongoing community trial to rapidly evaluate school responses to COVID-19. Other study strengths include the ability to engage remotely with administrators and educator citizen scientists to understand challenges during the COVID-19 pandemic, and inform timely decision-making processes using integrated knowledge translation. The study also amplifies the voices of educators from all teaching grades in informing future policy formulation. The study captured only school administrator and educator perspectives, and would benefit from gathering student, caregiver and family perspectives to inform ongoing strategies for school safety, online learning, and lockdown response.

## 5. Conclusions

This is the first study to evaluate school policies using digital citizen science approaches for understanding the impact of COVID-19 restrictions on educator and youth health in rural Indigenous communities. The findings revealed significant mental health concerns among students as well as educators, indicating the critical need for long-term strategies to build mental health support services. The lack of adequate infrastructure, healthcare access, and digital connectivity in rural Indigenous communities exacerbated the negative impact of COVID-19 on school health.

While this study identified significant overlap between administrator and educator perceptions, there were discrepancies with regards to rapid decision-making during the early days of the pandemic. Use of the Smart Platform enabled knowledge translation of this evidence to school leadership teams—an example of rapid research response using citizen science-based digital epidemiological platforms. This approach can be replicated in other school boards and jurisdictions worldwide to respond to not only COVID-19, but also other communicable and non-communicable diseases, particularly in rural and remote populations.

In conclusion, this study provides insight into using digital citizen science approaches to rapidly respond to societal crises such as the COVID-19 pandemic, and highlights the potential of digital health interventions and culturally-appropriate curricula to mitigate school mental health crises.
