# Supplementary material for: Preserving rural school health during the COVID-19 pandemic: Indigenous citizen scientist perspectives from a qualitative study
Source: AIMS Public Health. 2022 Jan 6;9(2):216–36. doi: 10.3934/publichealth.2022016 (PMC9114787; doi:10.3934/publichealth.2022016)
Supplement: Supplementary file 1 [file publichealth-09-02-016-s001.pdf]

AIMS Public Health

AIMS Public Health, 9(2): 216–236.
DOI: 10.3934/publichealth.2022016
Received: 29 October 2021
Revised: 24 December 2021
Accepted: 27 December 2021
Published: 06 January 2022

http://www.aimspress.com/journal/aimsph

Research article

Preserving rural school health during the COVID-19 pandemic: indigenous citizen scientist perspectives from a qualitative study

Prasanna Kannan[1], Jasmin Bhawra[2], Pinal Patel[1] and Tarun Reddy Katapally[3,*]

[1] Johnson Shoyama Graduate School of Public Policy, University of Regina, 2155 College Ave, Regina, SK S4P 4V5, Canada
[2] Johnson Shoyama Graduate School of Public Policy, University of Saskatchewan, 101 Diefenbaker Pl, Saskatoon, SK S7N 5B8, Canada
[3] Faculty of Health Sciences, Western University, 1151 Richmond St, London, ON N6A 5B9, UK

* **Correspondence:** Email: tkatapa@uwo.ca; Tel: +15196614249.

Appendix 1

SIY semi-structured interview guide for administrators

September 2020

Self – Introduction with name and designation

- *This discussion is to understand challenges during the COVID-19 lockdown and restrictions, and to understand steps been taken for safe school reopening.*
- *Your identity will be kept confidential and you can speak freely. This session is being recorded for research purposes, so if you feel uncomfortable you can turnoff your camera.*

COVID-19 lockdown challenges:

1. When did the school lock down?
2. How did you cope with the lockdown?

Probe: What changes did you make to your teaching?

3. What was the biggest challenge?
4. What programs suffered the most due to lockdown, apart from basic curricula?
5. Was there any land-based education during the lockdown?
6. How did students react to the lockdown?

Probe: Did you notice any changes in their anxiety levels? How was their general response to

online learning (if the school did online learning)?

7. How did educators react to the lockdown?
8. Considering the fact that the term had not ended, what steps did the school take to complete the school term?

Probe: How were the educators' and students' experiences with remote/online teaching?

Probe: What are the pros and cons of remote/online teaching?

9. What would you do differently if the school were to go into lock down again?

COVID-19 school reopening

Considering the safe return of students and educators to the schools,

1. How were the safe school re-opening policies and programs developed?

Probe: Can you take me through the processes that were involved in policy and program development? For instance, who were the key stakeholders? What was were the primary criteria? What were the biggest concerns?

2. What were the barriers or challenges in policy development?
3. Were you able to address these challenges?

Probe: If yes, how? If no, what do you think were the ramifications of these challenges?

4. Were any steps taken to bring educators and parents on board with the policy and program development for safe school re-opening?

Probe: What was the educator and parental response?

5. What do you think are the strengths and weaknesses of these policies and programs?

Probe: What do you like about them? What could be improved?

6. How do you think school reopening will affect students and educators?

Probe: Do you feel anxious about your own health or your student's health? What worries you?

7. What do you think are challenges for safe school reopening?
8. In an ideal scenario, how would you have developed and implemented these policies?
9. What do you think is the future of digital curricula and land-based learning during the COVID-19 era?

Probe: How do you think COVID has influenced online curriculum? How has it affected land-based learning?

AIMS Press

© 2022 the Author(s), licensee AIMS Press. This is an open access article distributed under the terms of the Creative Commons Attribution License (http://creativecommons.org/licenses/by/4.0)
