# Supplementary material for: Preserving rural school health during the COVID-19 pandemic: Indigenous citizen scientist perspectives from a qualitative study
Source: AIMS Public Health. 2022 Jan 6;9(2):216–36. doi: 10.3934/publichealth.2022016 (PMC9114787; doi:10.3934/publichealth.2022016)
Supplement: Supplementary file 2 [file publichealth-09-02-016-s002.pdf]

AIMS Public Health

AIMS Public Health, 9(2): 216–236.
DOI: 10.3934/publichealth.2022016
Received: 29 October 2021
Revised: 24 December 2021
Accepted: 27 December 2021
Published: 06 January 2022

http://www.aimspress.com/journal/aimsph

Research article

Preserving rural school health during the COVID-19 pandemic: indigenous citizen scientist perspectives from a qualitative study

Prasanna Kannan[1], Jasmin Bhawra[2], Pinal Patel[1] and Tarun Reddy Katapally[3,]*

[1] Johnson Shoyama Graduate School of Public Policy, University of Regina, 2155 College Ave, Regina, SK S4P 4V5, Canada
[2] Johnson Shoyama Graduate School of Public Policy, University of Saskatchewan, 101 Diefenbaker Pl, Saskatoon, SK S7N 5B8, Canada
[3] Faculty of Health Sciences, Western University, 1151 Richmond St, London, ON N6A 5B9, UK

* **Correspondence:** Email: tkatapa@uwo.ca; Tel: +15196614249.

Appendix 2

Smart Indigenous Youth - Focus group template for educators

September 2020

Self – Introduction with name and designation

SIY Project overview (Video/ ppt)

- *This discussion is to understand challenges during COVID-19 lockdown and restrictions, and to understand steps been taken for safe school reopening.*
- *The identities of the participants will be kept confidential and participants can speak freely. This session is been recorded for research purposes, so if anyone feels uncomfortable they can turnoff their cameras.*

COVID-19 lockdown challenges:

1. When did the school locked down?
2. How did you cope with the lockdown?
   Probe: What changes did you make to your teaching?
3. What was the biggest challenge?
4. What was the students' reaction to the lockdown?

Probe: Did you notice any changes in their anxiety levels? How was their general response to online learning (if the school did online learning?)

5. Considering the fact that the semester had not ended, what steps did the school take to overcome those challenges during the lockdown?
   Probe: How was your experience with remote teaching/online teaching?
6. What would you do differently if the school were to go in to lock down again?

COVID-19 School reopening

Considering the safe return of students and educators to the schools,

1. Are you aware of school reopening policies or programs?
2. What do you think about these policies in general?
   Probe: what would you like about them? What could be improved?
3. How do you think school reopening will affect students and educators?
   Probe: Do you feel anxious about your own health or your student's health. What worries you?
4. What do you think are challenges for safe school reopening?
5. Are there any suggestions you would like to make in addressing the concerns in a safe school reopening plan?

AIMS Press

© 2022 the Author(s), licensee AIMS Press. This is an open access article distributed under the terms of the Creative Commons Attribution License (http://creativecommons.org/licenses/by/4.0)
